# Validation of Saliva as the Clinical Specimen Type for a University-Wide COVID-19 Surveillance Program

**DOI:** 10.3390/v16091494

**Published:** 2024-09-21

**Authors:** Michael L. Farrell, Anton V. Bryksin, Emily Ryan, Jessica Lin, Naima Djeddar, German Khunteev, Benjamin Holton, Miles Paca, Nicholas Speller, James T. Merrill, Ted M. Ross, Robert J. Hogan, Greg Gibson, Andrés J. García, Michael P. Shannon

**Affiliations:** 1 Advanced Concepts Lab, Georgia Tech Research Institute, Atlanta, GA 30318, USA; 2 Petit Institute for Bioengineering and Bioscience, Georgia Institute of Technology, Atlanta, GA 30332, USA; 3 Stamps Student Health Services, Georgia Institute of Technology, Atlanta, GA 30332, USA; 4 Animal Health Research Center, Department of Infectious Diseases, University of Georgia, Athens, GA 30602, USA; 5 School of Biological Sciences, Georgia Institute of Technology, Atlanta, GA 30332, USA; 6 School of Mechanical Engineering, Georgia Institute of Technology, Atlanta, GA 30332, USA; 7 Office of the President, University of North Georgia, Dahlonega, GA 30597, USA

**Keywords:** saliva, nasopharyngeal swap, COVID-19, SARS-CoV-2, RNA, surveillance

## Abstract

At the beginning of the COVID-19 pandemic, the Georgia Institute of Technology made the decision to keep the university doors open for on-campus attendance. To manage COVID-19 infection rates, internal resources were applied to develop and implement a mass asymptomatic surveillance program. The objective was to identify infections early for proper follow-on verification testing, contact tracing, and quarantine/isolation as needed. Program success depended on frequent and voluntary sample collection from over 40,000 students, faculty, and staff personnel. At that time, the nasopharyngeal (NP) swab, not saliva, was the main accepted sample type for COVID-19 testing. However, due to collection discomfort and the inability to be self-collected, the NP swab was not feasible for voluntary and frequent self-collection. Therefore, saliva was selected as the clinical sample type and validated. A saliva collection kit and a sample processing and analysis workflow were developed. The results of a clinical sample-type comparison study between co-collected and matched NP swabs and saliva samples showed 96.7% positive agreement and 100% negative agreement. During the Fall 2020 and Spring 2021 semesters, 319,988 samples were collected and tested. The program resulted in maintaining a low overall mean positivity rate of 0.78% and 0.54% for the Fall 2020 and Spring 2021 semesters, respectively. For this high-throughput asymptomatic COVID-19 screening application, saliva was an exceptionally good sample type.

## 1. Introduction

At the beginning of the pandemic during the summer of 2020, institutions of higher education faced the difficult decision of whether to remain open for the upcoming fall semester and conduct classes in-person, switch completely to online classes, or some hybrid combination. Georgia Tech Research Institute (GT) decided to remain open to continue to offer its highest quality of education. To keep the 40,000 students, faculty, and staff safe, beyond the standard masking and social distancing, GT developed and implemented a high-throughput asymptomatic COVID-19 surveillance program. The objective of this program was to minimize new infections as much as possible by the early diagnosis of SARS-CoV-2 infections followed by patient notifications, contact tracing, and patient quarantine/isolation as recommended [1]. In retrospect, this decision was prescient as we now know through contact tracing studies and modeling that 15–25% of infections are asymptomatic and that asymptomatic people do transmit infection [2,3].

To establish the program, GT set up a Clinical Laboratory Improvement Amendments (CLIA)-certified laboratory with the capacity to process and test up to 1500 clinical samples per day and report results within 12 h after sample collection. The system design would allow people to test multiple times per week and meet the predicted testing frequency needed according to published reports and modeling [4,5]. Volunteer sample collection was made convenient through six collection sites proximate to residential areas and central campus hubs. Mobile pop-up collection sites were also instituted for special events as needed. Participation was encouraged through targeted messaging, signage, verbal referrals, and food-treat incentives. Collection sites were operational generally Mondays through Fridays throughout the semesters.

To be successful, the entire endeavor hinged on voluntary and frequent sample submissions. At that time, the main clinical sample type accepted for coronavirus diagnostics was the nasopharyngeal (NP) swab. However, because of the discomfort and the need to be collected by trained personnel, the NP swab did not lend itself to voluntary and frequent sample collection [6]. Additionally, the demand for NP swabs skyrocketed, creating a significant supply shortage of unknown duration and placing an even greater emphasis on the need for an alternative sample type. Saliva was the obvious alternative sample of choice for its ease of self-collection, but recently published reports showed mixed results, with either poorer [7,8,9,10], equivalent [11,12,13,14,15,16], or superior [17] performance as compared to the NP swab for detecting SARS-CoV-2 RNA. 

In order to substitute saliva for the NP swab as the input sample type for the COVID-19 diagnostic test, we developed and validated a sample collection kit and protocol. Next, we collaborated with the Georgia Department of Public Health (GDPH) and performed a comparative performance study on 522 matched co-collected NP swabs and saliva samples from actual human subject volunteers at a GDPH COVID-19 testing station. The results of these validation studies are presented here. 

## 2. Materials and Methods

### 2.1. Saliva Sample Collection

Commercial saliva collection devices were available, but the cost and potential supply chain issues led us to develop our own saliva collection device (see Supplemental Figure S1 for device component details and collection instructions). The collection kit components were assembled from low-cost, readily available items that were locally sourced, if possible. For example, the collection, transport, and storage tubes preloaded with six 2.8 mm zirconium beads were purchased in bulk from OMNI International, Kennesaw, GA, USA. The collection kit components were assembled into kits with the assistance of an XL100 automated liquid handler (BioMicroLabs, Concord, CA, USA), which dispensed 250 µL of collection buffer (either DNA/RNA Shield (Cat# R1100; Zymo Research, Irvine, CA, USA) or TNA buffer (Cat# TNA-1000; Omega Bio-tek, Norcross, GA, USA) into a saliva collection tube. 

### 2.2. Saliva Sample Clinical Validation Study

In collaboration with the Georgia Department of Public Health (GDPH), 522 matched NP swabs and saliva samples were co-collected from volunteers at a Public Health drive-thru testing station. The NP swab collection was administered by a trained GDPH nurse, while the saliva sample was self-collected by the volunteer using the GT collection kit under GT supervision. After collection, saliva samples were kept at 4 °C during storage and transport to the GT CLIA lab and frozen at −20 °C until processing and analysis. GDPH processed and analyzed the NP swabs using Hologic’s Panther System. Other details of the GDPH NP swab sample processes were not made available to us. GDPH provided NP swab analysis results as either “Negative” (437 samples), “Undetermined” (2 samples), or “Positive” (83 samples) for the presence of SARS-CoV-2. No Ct values were provided with the GDPH analysis results. To meet minimum comparative sample number requirements, GT’s CLIA lab selected matched saliva samples corresponding to 30 positive and 30 negative GDPH NP swab samples and processed and analyzed them as singlets using the GT-developed methods described here in both the 96-well (25 μL/reaction) and 384-well (10 μL/reaction) formats. 

### 2.3. Saliva Sample Processing and Pooling

Once samples were received and passed visual inspection, they were pre-processed in preparation for RNA extraction. We observed a surprising variety of appearances, colors, and viscosities of saliva collected from different individuals—indeed, even from the same individual at different times of the day. High viscosity posed a significant challenge for the automated liquid handlers, increasing the risk of cross-contamination. To address this issue, we employed OMNI’s Bead Ruptor Elite and collected samples in their compatible 2 mL reinforced polypropylene tubes containing six 2.8 mm zirconium beads. Different bead sizes, numbers, and materials were tested for optimal performance with saliva samples. Up to 24 single sample tubes can be loaded evenly spaced on the perimeter of the Bead Ruptor’s circular sample holding plate. This ensured that all samples experienced the same degree of bead disruption. This is in comparison with other homogenization devices that require loading multiple samples into rectangular sample holding devices in which wells on the outside can experience more bead disruption than those on the inside, resulting in uneven sample liquification. In our system, the Bead Ruptor’s rapid shaking and multi-plane orbital rotation (45 s at 6 m/s) provided even and superior liquification and homogenization of every saliva sample. Following bead-beating, the samples were briefly centrifuged.

Next, the samples were batched into a group of 225, scanned into LIMS, and arrayed into a 96-deep-well Kingfisher extraction plate by an Assist Plus robotic liquid handler (Integra Biosciences, Hudson, NH, USA) located inside a biosafety cabinet. The samples were arrayed according to a GT-developed 5x double pooling strategy [18] to a total volume of 200 µL. This pooling strategy provided a two-fold increase in our sample throughput and reduced our reagent costs. 

### 2.4. RNA Extraction

RNA extractions were performed in a designated space. Per sample, the RNA extraction buffer consisted of 240 µL TNA buffer, 8 µL Carrier RNA, and 280 µL isopropanol. This was prepared as a master mix, of which 528 µL was added to each 200 µL sample. RNA was then purified using the KingFisher Flex Magnetic Particle Processor (Thermo Scientific, Waltham, MA, USA) and the Mag-Bind Viral DNA/RNA 96 Kit (Omega-Bio-tek, M6246, Norcross, GA, USA) and eluted in 100 µL of nuclease-free water. 

### 2.5. Genomic RNA Quantification

Purified SARS-CoV-2 genomic viral RNA was quantified by copy number via digital droplet PCR (BioRad, Hercules, CA, USA) following the manufacturer’s instructions.

### 2.6. RT-PCR

Testing utilized the OPTI SARS-CoV-2 RT-PCR Test kit (IDEXX Laboratory, Westbrook, ME, USA). This kit utilized the FDA Emergency Use Authorization (EUA)-approved primers and probes targeting the SARS-CoV-2 N1 and N2 genes, as designed and validated by the Centers for Disease Control and Prevention [19]. The kit also contains primers and probes targeting the human RNase P gene as a human clinical sample positive control. RT-PCR kit reagents were mixed and assembled in a designated space and then transferred to a different space for sample addition. Following the manufacturer’s instructions, RT-PCR reagents were added to samples either manually by multichannel pipette to a 96-well plate or automatically to a 348-well plate by the ECHO acoustic liquid handler (LABCYTE, San Jose, CA, USA). RT-PCR was performed and analyzed on the ThermoFisher QuantStudio 6 FLEX platform. The double pooling scheme allowed for the direct identification of the specific sample responsible for any RT-PCR-positive pooled sample. This sample result was verified by repeat extraction and testing as a singlet using the original sample material for diagnostic purposes.

## 3. Results

### 3.1. NP-Swab vs. Saliva Sample Clinical Validation Study

Of the 522 GDPH NP swab sample analysis results (437 negative, 83 positive, and 2 inconclusive), the GT CLIA Lab randomly selected matched saliva samples that correlated to 30 positive and 30 negative NP swab samples. Thirty was selected as the minimum number of comparators required for the FDA’s pre-EUA application. These comparison results showed 96.7% positive/100% negative agreement (96-well, 25 μL/reaction) and 93.3% positive/100% negative agreement (384-well format, 10 μL/reaction), respectively (see Supplemental Table S1).

### 3.2. Sample Collection Buffer Validation

For the study described above, after collection, the NP swab samples were stored and transported in viral transport media (VTM). To be as consistent as possible, we also collected the study saliva samples in VTM as well. However, for biosafety considerations, we wanted a collection buffer that would inactivate the virus directly upon collection. We selected two alternative products for validation, DNA/RNA Shield (Zymo Research, Irvine, CA, USA) and TNA buffer (Omega, Bio-tek). The inactivation of SARS-CoV-2 by these two products was demonstrated under BSL-3 biosafety conditions by our collaborators at the Animal Health Research Center, University of Georgia. Live SARS-CoV-2 virus spiked into saliva was completely inactivated in a 50/50 mix with either of these two products. 

We performed a bridging study between these two products and VTM to test for suitability as sample input into our processing and analysis workflow. Live SARS-CoV-2 RNA virus (kindly provided by Jeff Hogan, UGA) was spiked into each of three sets of 10 tubes, each containing 250 μL of SARS-CoV-2-negative pooled human saliva samples (LeeBio, Maryland Heights, MO, USA). Each set then received 250 μL per sample of either of the three collection buffers followed by processing and analysis. The resulting Ct and standard deviation for each set were as follows: VTM, 25.77 ± 0.17; DNA/RNA Shield, 25.71 ± 0.08; and TNA, 25.39 ± 0.08 (see supplemental data Table S2) demonstrating performance equivalency as a suitable alternative saliva collection buffer. 

### 3.3. Assay Limit of Detection

The assay limit of detection (LOD), using purified target RNA spiked directly into RT-PCR reactions, was reported by the CDC as five target copies per 5 μL of the sample [19]. To determine the assay limit of detection in our hands, we spiked three independent sets of twenty replicate RT-PCR reactions (20 μL each) with 5 μL each of water containing either 10 or 25 copies of SARS-CoV-2 genomic RNA. We achieved 48/60 positive results against 10 genome copies and 60/60 positive results against 25 copies. Thus, in our hands, the conservative assay limit of detection is 25 target copies per 5 μL of purified target RNA (see Supplemental Tables S2 and S3). 

### 3.4. Process Limit of Detection

As opposed to the RT-PCR assay limit of detection with clean reagents, the process limit of detection takes into account the entire workflow to include the physical properties of the organism, the sample matrix with potential RT-PCR inhibitors, sample transport and storage, and nucleic acid losses that occur during the sample extraction process. To determine the process limit of detection, we spiked two sets of 60 replicate samples containing 250 μL of pooled human saliva with either 500 or 1000 genome copies of the SARS-CoV-2 virus. At 500 spiked genomes, we detected 43/60 positive samples, and at 1000 copies, we detected 60/60 positive samples (see supplementary data Table S4). Thus, our process limit of detection is stated as 1000 SARS-CoV-2 genomes. We acknowledge the limitation of determining process LOD with purified genomic RNA rather than actual virus measured in plaque-forming units due to biosafety restrictions.

### 3.5. Saliva Sample Pooling

**Figure 1:**
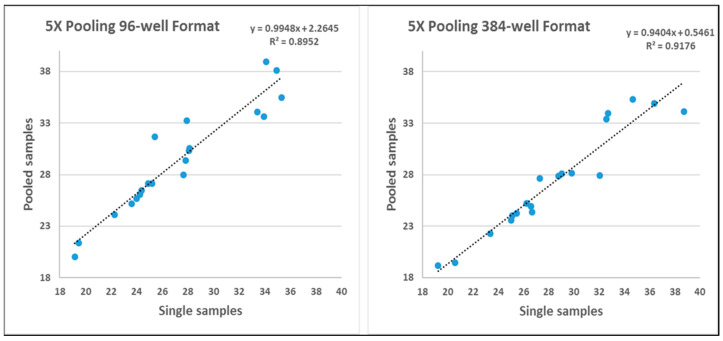
Regression analysis comparison of Ct values between a single saliva sample vs. 5x double pooled saliva samples in both the 96-well and 384-well plate formats.

As compared to single sample analysis, pooling dilutes the constituent samples, which increases the chances of false negatives. This disadvantage is weighed against increased throughput and lower costs. In analyzing 20 replicate samples as singlet vs. 5x double pooled, we observed an average drop of 2.12 ± 1.68 Cts for the 96-well plate format and 1.32 ± 1.72 Cts for the 384-well format (Figure 1). It was curious that the Ct drop for pooled samples was less for the 394-well format, which contains lower sample constituent volumes (0.4 μL), than for the 96-well format (1.0 μL). This may be due to the increased accuracy of the Echo acoustic liquid handler in loading the 394 plate vs. manual reagent addition of the 96-well plate using a multi-channel pipettor. Alternatively, it has been suggested that the optical imaging system of the QuantStudio 6 FLEX platform may better discriminate signal acquisition from a 384-well layout.

The regression analysis shows that pooling saliva samples in both 96-well and 384-well formats maintains a predictable relationship with the Ct values observed in single samples, thereby supporting the validity of using pooled samples for diagnostic purposes. This validation is crucial as it ensures that pooling does not significantly compromise the sensitivity and specificity of the assay, which is especially important in high-throughput settings or where sample conservation is necessary. We are not sure what coefficient the reviewer is referring to, though. 

For the slope (b), which is approximately 0.9, the value suggests a slight systematic reduction in Ct values in pooled samples compared to single samples. This is most likely due to dilution effects or differences in sample processing between single and pooled samples. It is critical to note that the slope being close to 1 indicates that the increase in Ct values from single to pooled samples is almost proportional, suggesting that pooling does not dramatically alter the detection capability. 

For the y-intercept in the 96-well format, the value is 2.2465, and in the 384-well format, it is 0.5461. This difference shows that at the baseline (where x = 0), pooled samples in the 384-well format start with a lower Ct value relative to the 96-well format. This intercept difference could stem from differences in sample handling, well volume, Q-PCR instrument optics, or other procedural nuances between the two plate types. 

The coefficient of determination (R^2^) values (0.8952 for 96-well and 0.9176 for 384-well) indicate a high degree of linear correlation between the Ct values of single and pooled samples. High R2 values suggest that pooling does not significantly affect the detectability of the analyte, as most of the variation in pooled sample Ct values can be predicted from the single sample Ct values. 

Details of how the sample pooling was designed are presented elsewhere [18]. Briefly, each sample is represented twice in two separate wells, each in the context of four other different unique samples. This approach tests each sample in different analytical contexts and allows for the effective identification of the causative sample through cross-referencing constituent samples between two positive pools. If only one well of a plate is positive, the positive sample is likely near the LOD, and each constituent sample must be individually tested for verification.

## 4. Discussion and Conclusions

The successful results of the GDPH NP swab/saliva comparison study and other saliva validation experiments presented here were later substantiated by a systematic retrospective review [20] of similar sample comparison studies, confirming the excellent discriminatory and diagnostic ability of saliva and recommending it for mass screening applications. 

Saliva is a chemically complex sample type containing a variety of electrolytes, digestive enzymes, and immunoglobulins. In addition, saliva is likely to contain complex environmental substances from recently consumed food and beverages. The physical aspects of saliva as a sample are also something to contend with. The presence of mucus results in a viscous, non-homogenous specimen that is not suited for robotic liquid handling. To solve this, the saliva samples were collected in a reinforced 2 mL polypropylene tube with an O-ring screw cap preloaded with six 2.8 mm zirconium beads by OMNI for use on their bead mill homogenizer, Bead Ruptor Elite. This homogenization step consists of vigorous shaking of the collection tubes while simultaneously rotating around an orbital plane. This pre-extraction step completely liquifies and homogenizes the sample for maximum analyte exposure to the extraction reagents. Further demonstration of the effectiveness of this method is the validation of the 5x double pooling strategy in the 384-well format, which only contained 0.4 µL of each constituent sample. The Bead Ruptor Elite was also described as a key step in the SalivaAll protocol developed by our collaborators at August University, Augusta, GA, USA [21]. 

To help overcome the severe supply chain shortages, we locally sourced system supplies and reagents wherever possible. As previously mentioned, the OMNI collection tubes and bead mill homogenizer were locally sourced from their location in nearby Kennesaw, GA, USA. The critical viral RNA magnetic bead extraction reagents were procured from Omega Bio-tek in nearby Norcross, GA, USA. The steady supply of the critical OPTI SARS-CoV-2 RT-PCR test kits was facilitated by a local IDEXX distributor also in Norcross, GA, USA. The supply for critical pipette tips for the Integra Assist Plus liquid handlers became so constrained that we purchased and installed two tip washing machines (Grenova, Richmond, VA, USA) and validated tip reuse up to twenty times. Additionally, we validated tip-less instruments for plate reagent loading, such as the Echo 525 Acoustic (Beckman Coulter, Brea, CA, USA) and Mantis (Formulatrix, Dubai, UAE) liquid handlers. We also validated backup reagents as insurance against future shortages; for example, our BSL-3 collaborators at the University of Georgia validated two different collection buffers to replace VTM, DNA/RNA Shield, and TNA buffers for their ability to completely inactivate SARS-CoV-2 virus.

The 5x double pooling of saliva samples provides a high throughput and cost-effective method of analyzing many samples simultaneously with minimal risk of false negatives. By comparing the positive results of differently pooled samples, this strategy allowed for the direct identification of the specific positive sample in a pool rather than having to repeat test each constituent sample. This allowed for more timely identification of infected individuals, which reduced transmission probabilities and infection rates. 

**Figure 2:**
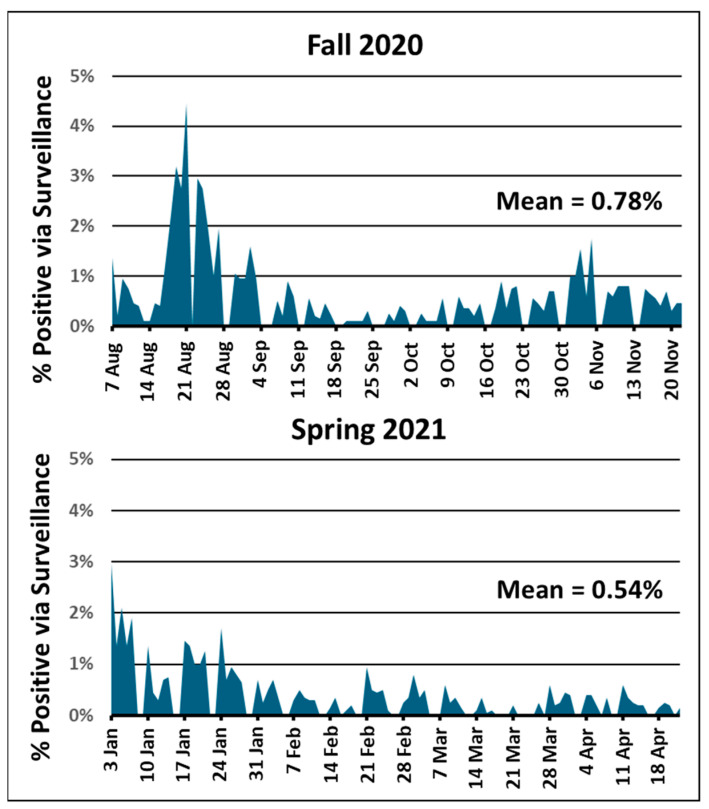
Surveillance positivity rate per day throughout the Fall 2020 and Spring 2021 semesters. Daily sample collection and testing were usually performed daily Monday through Friday. The mean positivity rate throughout each semester is indicated.

The results of the validation studies presented here were used to prepare and submit Pre-Emergency Use Authorization applications to the Food and Drug Administration for a Medical Device Manufacturer for the production of the GTRI Diagnostic Saliva Collection Kit (EUA202482) and a CLIA Laboratory-Developed Diagnostic Test (EUA202562) for processing and analyzing saliva for the detection of SARS-CoV-2 RNA. The submission of the EUA applications allowed us to operationalize the COVID-19 asymptomatic surveillance program and begin testing on August 7, 2020. This program kept the average positivity rate to 0.78% for the Fall 2020 semester and 0.54% for the Spring 2021 semester [18] (Figure 2). The lower rate for the Spring 2021 semester may be due to the introduction in December 2020 of the first vaccine for persons 16 years or older.

Figure 2 shows that the highest positivity rates were during the first three weeks of both the Fall 2020 and Spring 2021 semesters. These high positivity rates were likely the result of off-campus exposures during the preceding summer and winter breaks being imported onto campus. Once students were back in the GT testing environment, the positivity rates settled down. If a certain component of the campus community was experiencing a high positivity rate, for example, fraternities or sororities vs. residential dormitories, the sample collection stations were moved to be more concentrated in that community. A statistical breakdown of testing participation and results from the various components of the GT campus community are presented elsewhere [18]. The results presented here provided early evidence supporting the use of saliva as a viable sample type for mass surveillance for SARS-CoV-2 infection.

## Data Availability

Any data supporting this study’s findings that are not presented here are available from the corresponding authors upon reasonable request.

## Supplementary Materials

The following supporting information can be downloaded at: https://www.mdpi.com/article/10.3390/v16091494/s1, Figure S1: saliva collection kit; Table S1: RT-PCR results of GT saliva samples corresponding to 30 negative and 30 positive NP swab samples from GDPH analysis, Table S2: Triplicate sets of 20 RT-PCR reactions spiked with 10 copies of SARS-CoV-2 viral RNA, Table S3: Triplicate sets of 20 RT-PCR reactions spiked with 25 copies of SARS-CoV-2 viral RNA, Table S4: Saliva sample processing and analysis limit of detection. Table S5. Ct comparison between single and 5X sample pooling in 96 and 384 well formats.
