# Supplementary material for: Validation of Saliva as the Clinical Specimen Type for a University-Wide COVID-19 Surveillance Program
Source: Viruses. 2024 Sep 21;16(9):1494. doi: 10.3390/v16091494 (PMC11437455; doi:10.3390/v16091494)
Supplement: Supplementary file 1 [file viruses-16-01494-s001.zip › viruses-3168703-supplementary.pdf]

Supplemental Data.

Collection kit and method description.

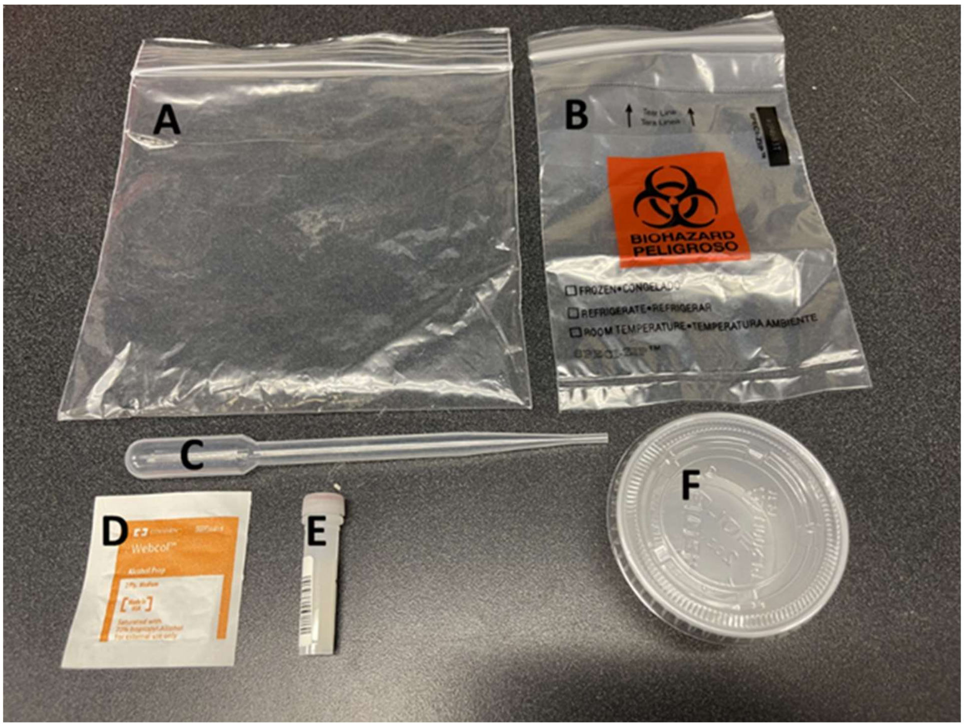

Supplemental Figure S1. Saliva collection kit. A. Kit container zip lock bag; B. Hazardous waste zip lock bag; C. Transfer pipette; D. Alcohol wipe; E. Barcoded sample transport and storage tube preloaded with six 2.8 mm ceramic beads and 250 μL of either DNA/RNA Shield (Zymo Research, Irvine, CA) or TNA Buffer (Omega Bio-tek, Norcross, GA); F. Plastic specimen collection cup with lid.

The saliva sample self-collection method started as each asymptomatic volunteer registered their sample submission through a GT-developed smart phone app. This app tied directly into the GT-developed laboratory information management system (LIMS) which tracked the sample by its barcode all the way through processing, analysis, and results. Sample collection was performed under supervision with the volunteer following printed instructions as follows:

1. Open zip lock bag (Figure 1.A) containing kit components and empty contents onto provided clean table surface.

2. Remove lid from specimen collection cup (Figure 1.F). Spit into the specimen collection cup repeatedly to provide a volume of saliva that completely covers the bottom of the cup.

3. Remove cap from the collection tube (Figure 1.E) and use the plastic pipette (Figure 1.C) to transfer saliva from collection cup to the collection tube.

4. Transfer a sufficient amount of saliva (~400 μL) to the sample collection tube to meet the fill line printed on the label. If the fill line is not met, continue spitting into the cup and perform additional transfers with the pipette until meeting the fill line. The sample collection tube is preloaded with six 2.8 mm ceramic beads and 400 μL of either DNA/RNA Shield (Zymo Research, Irvine, CA) or TNA Buffer (Omega Bio-tek, Norcross, GA).

5. Replace the cap onto the collection tube, close screw cap firmly, and shake tube several times to allow the beads inside the collection tube to mix the liquid sample.

6. Add first and last initials to the collection tube label using the provided laboratory marker.

7. Place the capped and labeled collection tube into the provided specimen bag (Figure 1.B) and seal.

8. Wipe down the specimen bag with the provided alcohol wipe (Figure 1.D).

9. The Collection Supervisor will then scan your sample registration barcode from the app and the barcode on the collection tube.

10. Place the specimen bag containing the collection tube into the provided bin at the collection site.

11. Place all other collection kit materials into the original collection kit zip lock bag, seal, and deposit into the collection hazardous waste bin.

Supplemental Table S1. RT-PCR results of GT saliva samples corresponding to 30 negative and 30 positive NP swab samples from GDPH analysis. Results are shown in both the 96- and 384-well formats.

96 well format						384 well format					
Known positive samples			Known negative samples			Known positive samples			Known negative samples		
	CT values		96	CT values		384	CT values		384	CT values	
Sample	N1,N2	Rnase P	Sample	SARS-CoV-2	Rnase P	Sample	N1,N2	Rnase P	Sample	SARS-CoV-2	Rnase P
1	30.92	29.15	31	Undetermined	28.68	1	30.60	28.39	31	Undetermined	27.68
2	Undetermined	36.53	32	Undetermined	32.27	2	Undetermined	34.38	32	Undetermined	30.64
3	30.37	38.18	33	Undetermined	30.40	3	30.94	34.72	33	Undetermined	29.21
4	27.84	28.97	34	Undetermined	34.04	4	26.83	27.81	34	Undetermined	33.54
5	19.48	Undetermined	35	Undetermined	33.91	5	18.69	Undetermined	35	Undetermined	33.06
6	32.39	35.55	36	Undetermined	30.16	6	32.04	34.99	36	Undetermined	28.99
7	28.07	39.88	37	Undetermined	34.72	7	27.16	33.45	37	Undetermined	32.99
8	33.94	31.92	38	Undetermined	28.84	8	34.98	30.77	38	Undetermined	27.86
9	24.37	33.89	39	Undetermined	32.37	9	23.49	32.54	39	Undetermined	30.93
10	29.62	35.72	40	Undetermined	31.12	10	28.56	33.52	40	Undetermined	29.79
11	24.92	34.86	41	Undetermined	34.66	11	24.41	29.46	41	Undetermined	33.44
12	25.43	27.27	42	Undetermined	29.95	12	24.34	25.96	42	Undetermined	28.79
13	34.95	36.16	43	Undetermined	33.40	13	33.44	34.58	43	Undetermined	32.56
14	27.64	33.42	44	Undetermined	29.34	14	26.66	32.00	44	Undetermined	28.52
15	19.18	30.71	45	Undetermined	31.00	15	18.56	28.80	45	Undetermined	29.73
16	29.22	34.09	46	Undetermined	31.50	16	29.05	34.13	46	Undetermined	30.28
17	28.15	33.62	47	Undetermined	31.80	17	27.62	32.20	47	Undetermined	30.86
18	28.87	33.66	48	Undetermined	30.65	18	27.99	32.54	48	Undetermined	29.93
19	24.28	Undetermined	49	Undetermined	33.92	19	23.67	39.09	49	Undetermined	33.33
20	38.62	34.24	50	Undetermined	33.26	20	Undetermined	32.83	50	Undetermined	32.23
21	32.54	29.27	51	Undetermined	34.78	21	32.10	28.30	51	Undetermined	34.94
22	23.60	37.83	52	Undetermined	28.95	22	22.48	27.81	52	Undetermined	27.81
23	34.13	31.30	53	Undetermined	30.76	23	33.48	30.53	53	Undetermined	29.41
24	35.31	35.65	54	Undetermined	40.71	24	36.95	33.99	54	Undetermined	31.95
25	25.22	32.77	55	Undetermined	34.46	25	24.22	30.77	55	Undetermined	33.95
26	24.01	28.28	56	Undetermined	36.05	26	22.93	26.77	56	Undetermined	33.37
27	33.40	33.43	57	Undetermined	31.26	27	32.97	33.03	57	Undetermined	30.39
28	22.29	44.19	58	Undetermined	32.57	28	21.71	Undetermined	58	Undetermined	33.28
29	29.24	29.78	59	Undetermined	34.69	29	28.70	28.60	59	Undetermined	34.93
30	27.90	38.23	60	Undetermined	35.55	30	26.88	34.17	60	Undetermined	35.17

N1, N2 = virus gene targets; Rnase P = control human gene target

Supplemental Table S2. Triplicate sets of 20 RT-PCR reactions spiked with 10 copies of SARS-CoV-2 viral RNA. Each sample (n=60) was extracted individually.

10 viral genome copies								
Set1	CT values		Set2	CT values		Set3	CT values	
	N1,N2	RP		N1,N2	RP		N1,N2	RP
1	34.18	33.85	1	34.69	33.71	1	Undetermined	34.43
2	35.05	33.25	2	Undetermined	34.15	2	36.02	33.77
3	38.48	36.07	3	34.23	34.57	3	Undetermined	35.02
4	34.55	34.02	4	34.39	34.13	4	34.62	33.62
5	Undetermined	34.24	5	37.66	34.71	5	34.2	35.04
6	36.64	35.75	6	35.92	34.96	6	36.02	34.66
7	35.75	33.79	7	Undetermined	34.02	7	34.81	34.97
8	36.15	33.72	8	36.93	34.59	8	36.49	33.86
9	Undetermined	34.73	9	36.35	34.29	9	35.33	34.84
10	36.08	33.99	10	Undetermined	34.36	10	Undetermined	33.66
11	Undetermined	35.2	11	35.38	34.7	11	36.99	35.08
12	34.26	33.76	12	35.18	35.21	12	34.69	33.47
13	35.5	35.78	13	38.17	33.92	13	36.17	35.29
14	34.72	34.54	14	35.02	34.47	14	35.68	33.42
15	34.57	33.98	15	35.82	34.81	15	35.44	35.09
16	37.65	35.03	16	34.99	33.88	16	35.09	34.06
17	35.51	34.36	17	Undetermined	35.56	17	35.14	35.61
18	33.48	33.59	18	36.87	33.86	18	36.11	34.35
19	36.06	33.97	19	Undetermined	34.43	19	Undetermined	35.26
20	35.78	35.18	20	36.87	33.86	20	35.92	34.48

N1, N2 = virus gene targets; RP = RNase P, control human gene target

Supplemental Table S3. Triplicate sets of 20 RT-PCR reactions spiked with 25 copies of SARS-CoV-2 viral RNA. Each sample (n=60) was extracted individually.

25 viral genome copies								
Set1	CT values		Set2	CT values		Set3	CT values	
	N1,N2	RP		N1,N2	RP		N1,N2	RP
1	33.54	33.88	1	34.07	34.01	1	34.28	33.29
2	33.79	33.27	2	32.68	33.44	2	33.64	33.29
3	33.21	33.54	3	34.04	33.63	3	33.34	33.1
4	33.53	33.25	4	33.86	33.97	4	32.82	33.36
5	33.85	34.15	5	33.38	33.14	5	34.76	32.9
6	33.07	33.3	6	33.33	33.41	6	33.24	33.33
7	33.13	33.57	7	34.7	33.79	7	33.12	32.73
8	32.91	33.43	8	33.68	33.27	8	32.47	33.29
9	33.18	33.47	9	33.13	33.48	9	32.55	33.74
10	31.36	33.34	10	32.81	33.6	10	33.11	33.65
11	32.75	33.72	11	33.04	33.64	11	33.64	33.71
12	32.53	33.09	12	33.28	33.32	12	33.04	33.83
13	32.19	33.36	13	33.51	33.84	13	32.73	33.58
14	32.61	33.33	14	33.51	33.13	14	32.84	33.68
15	33.74	33.59	15	33.51	33.61	15	33.12	33.65
16	32.96	33.24	16	33.51	33.27	16	33.82	33.5
17	33.27	33.46	17	33.51	32.63	17	33.09	33.03
18	33.41	33.37	18	33.51	33.1	18	32.04	33.45
19	33.52	33.13	19	33.88	33.42	19	32.97	33.08
20	32.53	33.09	20	34.68	33.38	20	33.3	33.27
Mean Ct ± SD for N1,N2 = 33.28 ± 0.62								

N1, N2 = virus gene targets; RP = RNase P, control human gene target

Supplemental Table S4. Saliva sample processing and analysis limit of detection.

500 viral genome copies								
set1	CT value		set2	CT value		set3	CT value	
	N1,N2	RP		N1,N2	RP		N1,N2	RP
1	Undetermined	28.39	1	37.53	29.02	1	38.23	28.45
2	34.00	28.86	2	34.77	28.95	2	34.98	29.70
3	Undetermined	29.18	3	Undetermined	28.81	3	39.13	28.79
4	37.74	29.41	4	36.06	28.44	4	36.66	28.54
5	36.74	28.27	5	36.73	28.15	5	36.41	28.04
6	37.13	28.74	6	35.80	28.59	6	Undetermined	28.74
7	37.31	28.44	7	38.64	28.49	7	37.98	28.40
8	36.73	28.49	8	37.90	28.50	8	38.07	28.47
9	35.60	28.22	9	36.03	28.22	9	35.88	28.20
10	39.76	29.10	10	39.32	29.12	10	37.76	29.11
11	39.24	28.72	11	37.08	28.80	11	37.66	28.71
12	36.65	28.61	12	36.72	28.99	12	37.26	28.61
13	Undetermined	28.33	13	36.96	28.87	13	37.03	28.33
14	Undetermined	28.70	14	Undetermined	28.42	14	Undetermined	28.44
15	36.06	28.15	15	37.24	28.12	15	37.02	28.10
16	38.49	28.33	16	38.64	28.39	16	37.76	28.82
17	Undetermined	Undetermined	17	Undetermined	Undetermined	17	Undetermined	Undetermined
18	33.98	26.17	18	34.15	26.11	18	33.10	26.14
19	36.55	28.41	19	Undetermined	28.75	19	Undetermined	28.37
20	38.51	28.54	20	Undetermined	27.88	20	38.34	28.57
1,000 viral genome copies								
	CT value			CT value			CT value	
set1	N1,N2	RP	set2	N1,N2	RP	set3	N1,N2	RP
1	35.08	34.48	1	36.85	34.21	1	36.75	34.31
2	31.98	31.56	2	32.81	31.53	2	32.32	31.44
3	33.97	31.96	3	33.19	32.10	3	32.92	31.82
4	32.72	32.40	4	31.98	32.42	4	32.34	32.79
5	31.14	30.52	5	30.75	30.38	5	30.59	30.38
6	34.94	32.47	6	34.35	32.52	6	34.79	32.41
7	32.34	32.09	7	32.29	32.16	7	33.18	32.42
8	31.59	32.63	8	31.57	32.87	8	31.15	32.63
9	34.03	32.29	9	33.35	32.38	9	33.13	32.39
10	36.76	34.70	10	38.01	35.06	10	37.47	34.79
11	34.39	33.69	11	34.02	33.47	11	33.14	33.69
12	35.44	32.51	12	34.92	32.91	12	35.71	32.58
13	35.01	33.23	13	35.71	33.76	13	35.36	33.65
14	34.00	32.98	14	34.02	32.88	14	33.01	32.53
15	33.10	31.84	15	33.05	31.54	15	34.14	31.84
16	34.24	33.14	16	34.63	32.98	16	34.08	32.72
17	33.57	32.59	17	33.88	32.46	17	33.07	32.68
18	33.43	31.63	18	33.48	31.73	18	32.98	31.42
19	33.54	32.53	19	34.03	32.99	19	32.88	32.77
20	35.03	32.19	20	33.99	31.92	20	33.16	31.80

N1, N2 = virus gene targets; RP = RNase P, control human gene target

Supplemental Table S5. Ct comparison between single and 5X sample pooling in 96 and 384 well formats.

96 well format			384 well format		
	Single	5X pool		Single	5X pool
Ct Ave	27.21	29.33	Ct Ave	28.56	27.21
Ct SD	4.92	5.18	Ct SD	5.02	4.92
ΔCt:	2.12 ± 1.68		ΔCt:	1.32 ± 1.71	
